# Neohesperidin Mitigates High-Fat-Diet-Induced Colitis In Vivo by Modulating Gut Microbiota and Enhancing SCFAs Synthesis

**DOI:** 10.3390/ijms26020534

**Published:** 2025-01-10

**Authors:** Kun Lu, Sijie Shan, Yanling Zeng, Guliang Yang

**Affiliations:** 1 National Engineering Laboratory for Rice and By-Products Processing, Food Science and Engineering College, Central South University of Forestry and Technology, Changsha 410004, China; lukun1986220@163.com (K.L.); 15974242363@163.com (S.S.); 2 Key Laboratory of Cultivation and Protection for Non-Wood Forest Trees, Ministry of Education, Central South University of Forestry and Technology, Changsha 410004, China; zengyanling110@126.com

**Keywords:** neohesperidin, intestinal microbiota, SCFAs, JAK2/STAT3 pathway, intestinal mucosa

## Abstract

Previous research has consistently shown that high-fat diet (HFD) consumption can lead to the development of colonic inflammation. Neohesperidin (NHP), a naturally occurring flavanone glycoside in citrus fruits, has anti-inflammatory properties. However, the efficacy and mechanism of NHP in countering prolonged HFD-induced inflammation remains unclear. In this study, rats on HFD were intragastrically administered (i.g.) with NHP for 12 consecutive weeks. Results indicate that this natural compound is effective in reducing colorectal inflammation at doses of 40–80 mg/kg body weight (BW) by i.g. administration, with significant decreases in inflammation markers such as TNF-α and IL-1β levels. It also improved intestinal mucosal tissue integrity and reduced HFD-stimulated colorectal inflammation via the JAK2/STAT3 pathway. Furthermore, intestinal microbiota sequencing results show that NHP intervention significantly downregulated the Firmicutes/Bacteroidetes ratio. This ratio is closely related to the preventive role in the context of glycolipid metabolism disorder. Compared with fecal cultures of rats from the HFD group, after 48 h in vitro fermentation, those from the NHP group had distinct microbiota composition and notably higher concentrations of SCFAs. Collectively, these observations suggest that 80 mg/kg BW NHP possesses biological activities in downregulating HFD-induced colorectal inflammation by regulating intestinal flora and promoting SCFAs formation.

## 1. Introduction

Existing research reveals that individuals who consume high-fat diets (HFDs) for extended periods of time often experience excessive accumulation of fat. This process is often accompanied by dysregulation of the gut microbiota and increased levels of inflammation [1]. The gut microbiota is a symbiotic ecosystem consisting of the host and a large number of gut microbes. The study of the impact of the gut microbiome on chronic conditions such as obesity associated with HFD consumption has recently emerged as an area of significant scientific interest [2]. Studies have confirmed that the gut microbiota is closely linked to dietary-induced obesity [3]. Feeding germ-free mice an HFD led to distinct alterations in glucose loading tolerance, insulin sensitivity, and obesity compared to their normally fed counterparts. This highlights the significant impact of diet on gut microbiota [4]. Transplanting the gut microbiota of obese mice into the guts of normal mice led to obesity in these previously healthy mice [5]. In addition, numerous studies on gut microbiota composition in obese individuals and gut microbiota transplantation experiments have also demonstrated the crucial role of the gut microbiota in the pathogenesis of obesity [6].

With advances in gut microbiota research, the notion that gut microbiota serves as a target for dietary effects on host health has gained widespread acceptance [7]. HFDs impose potential perturbation on the native microbial population and its function in the gut. The composition and function of the gut microbiota are an intricate biological mechanism that can significantly impact various host biological processes, including immune and inflammatory responses. The gut microbiome is capable of producing and metabolizing various compounds, which can affect intestinal barrier function and various signaling pathways. These changes can subsequently affect the host’s metabolic equilibrium and overall health status [8]. The intestinal barrier exhibits selective permeability, which prevents the invasion of pathogenic microorganisms, toxins, and other harmful substances. It also ensures the normal absorption of nutrients and electrolytes. Therefore, the integrity of the intestinal barrier directly affects gut homeostasis [9]. Once this balance is disrupted, it can affect intestinal mucosa barrier function [10]. Mechanistically, the intestinal barrier is closely related to tight junction protein expression, mucin content, and intestinal epithelial cell proliferation. Current research has confirmed that Mucin 2 (MUC2) is the predominant component of mucins. This protein has the activity of inhibiting the activation of downstream inflammatory responses mediated by activating IL-6/JAK/STAT3 signaling cascade [11,12]. Therefore, MUC2 is crucial for maintaining the integrity of the intestinal mucosal barrier [13].

In vivo, specific gut microbiota can convert dietary fiber into short-chain fatty acids (SCFAs), such as acetic acid, propionic acid, butyric acid, and others. These SCFAs are of great significance in maintaining gut health. They also play an essential role in regulating the immune system and exerting an influence on energy metabolism [14], promoting intestinal peristalsis, and enhancing intestinal barrier function [15]. Chronic inflammation causes gut dysbiosis characterized by decreased beneficial microorganisms and increased harmful bacteria. Concurrently, the level of SCFAs is significantly reduced [14].

Citrus flavonoids can stimulate the proliferation of beneficial bacteria, such as *Bifidobacterium* and *Lactobacillus*, which are able to ferment dietary fiber efficiently to produce SCFAs [16]. This category of compounds also exerts an inhibitory effect on the growth of harmful gut microbiota. Therefore, it helps maintain the balance of the gut microbiota community and facilitates the formation of SCFAs [17]. Neohesperidin (NHP) exerts its anti-inflammatory effect by inhibiting the activation of pro-inflammatory cytokines and modulating immune cell functions. Its antioxidant property is attributed to scavenging free radicals and reducing oxidative stress [18]. Scientific research [19,20] has verified that NHP is effective in alleviating the symptoms of ulcerative colitis induced by the administration of sodium dextran sulfate (DSS), and this activity is closely related to its ability to modulate gut microbiota composition. However, the precise mechanism by which it exerts its effectiveness against colitis, especially its influence on the gut microbiota, remains to be elucidated.

This study aimed to comprehensively investigate the mechanism by which NHP regulates gut microbiota and SCFAs synthesis to fill these knowledge gaps and provide new insights into the treatment of HFD-induced colitis. Specifically, this study evaluated the impact of intragastrically administered NHP on the biodiversity and predominance of the gastrointestinal microbiota, as well as inflammation in rats on an HFD for 12 consecutive weeks. In addition, the molecular mechanism by which NHP prevents HFD-induced colorectal inflammation was investigated, thus laying a theoretical foundation for the efficient utilization of NHP.

## 2. Results and Discussion

### 2.1. NHP Improved Lipid Metabolism Disorders in HFD Rats

**Figure 1:**
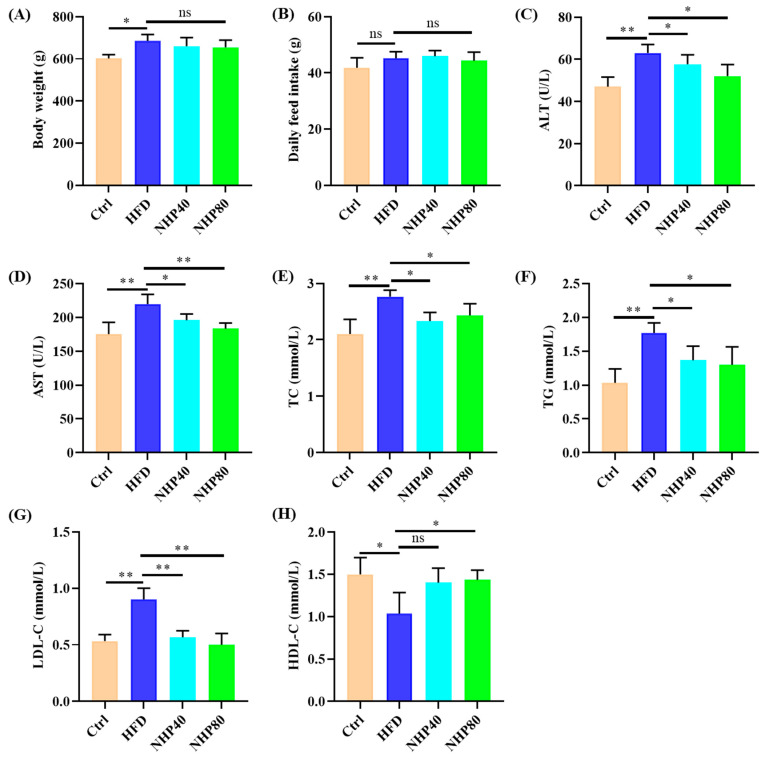
NHP mitigates obesity development in HFD rats. (**A**) Body weight at 12 weeks post-NHP treatment. (**B**) Daily food consumption. Serum parameters include (**C**) ALT, (**D**) AST, (**E**) TC, (**F**) TG, (**G**) LDL-C, and (**H**) HDL-C. Data are presented as mean ± SD (*n* = 3. * Indicates significance, * *p* < 0.05, ** *p* < 0.01, ns: no significant difference).

Figure 1A indicated that HFD rats exhibited markedly heavier body weights (BW, 684.8 g vs. 601.4 g, *p* < 0.05) compared to Ctrl rats. NHP treatment slightly reduced body weights (NHP40: 660.1 g, NHP80: 655.0 g, *p* > 0.05). Although food intake remained constant, it suggests that NHP’s effect on obesity is unrelated to reduced intake (Figure 1B). Figure 1C,D reveals significant elevations in serum ALT and AST activities (*p* < 0.01) in HFD rats compared to Ctrl (ALT: HFD > Ctrl, *p* < 0.01; AST: HFD > Ctrl, *p* < 0.01). Interestingly, NHP40 and NHP80 rats showed decreased serum ALT and AST activities (*p* < 0.05 or *p* < 0.01), indicating improved liver function. Figure 1E–H showed substantial increases in serum TC, TG, and LDL-C for HFD rats (*p* < 0.05 or *p* < 0.01) but decreased HDL-C levels (*p* < 0.01). NHP treatment effectively regulated these markers: serum TC, TG, and LDL-C levels were reduced (*p* < 0.05 or *p* < 0.01), and HDL-C levels were elevated (*p* < 0.05). In conclusion, these findings suggest that a chronic HFD induced lipid metabolism disorders in rats, which were effectively managed by NHP intervention. Long-term consumption of HFD can lead to dyslipidemia in the body. Specifically, levels of TC, TG, and LDL in the blood were found to increase. In addition, it can cause fat accumulation in the liver, resulting in fatty liver, and further lead to the elevation of ALT and AST [2]. Therefore, the above results demonstrate that NHP treatment can alleviate HFD-induced dyslipidemia and reduce fat accumulation in liver tissues.

### 2.2. NHP Improved Pathological Ileum Injury

**Figure 2:**
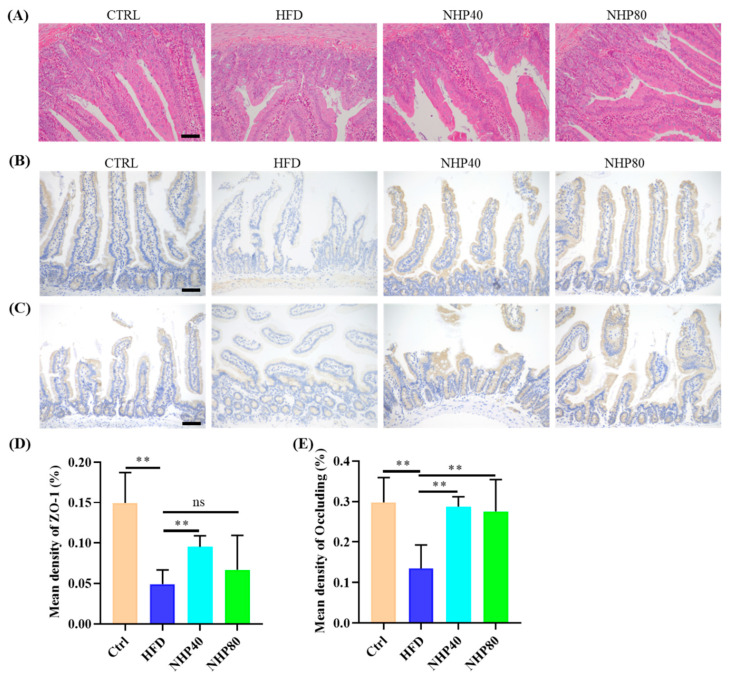
HE and IHC examination of intestinal tissue. (**A**) HE staining of intestinal tissues. In this HE-stained image, the cell nuclei appear bluish-purple (stained by hematoxylin), while the cytoplasm and extracellular matrix are pinkish (stained by eosin). (**B**) IHC of ZO-1 and (**D**) its quantification. (**C**) IHC of Occludin and (**E**) its quantification (200×). In these IHC images, brown areas represent positive expressions of ZO-1 and Occludin (visualized by DAB staining), and the cell nuclei are bluish-purple (counterstained by hematoxylin). The scale bar = 200 μm. Data are presented as mean ± SD (*n* = 3. ** *p* < 0.01, ns: no significant difference).

Hematoxylin and eosin (H&E) staining (Figure 2A) demonstrated that HFD induced abnormal proliferation of goblet cells, damage to lamina propria, and villous atrophy, thereby disrupting submucosal architecture. In contrast, NHP treatment not only effectively suppressed abnormal proliferation of goblet cells but also mitigated lamina propria damage and reversed villous atrophy. This led to the restoration of the submucosal architecture to a more normal state, as evidenced by the significant improvement in histological appearance compared to the HFD group. Compared to CTRL, immunohistochemical (IHC) staining revealed that HFD decreased levels of ZO-1 (Figure 2B,D, *p* < 0.01) and Occludin (Figure 2C,E, *p* < 0.01) in the ileum. NHP enriched the expression of ZO-1 (*p* < 0.01) and Occludin (*p* < 0.01), indicating enhanced intestinal tight junctions. These proteins significantly regulated cell polarity and apical domain development and impacted crucial cellular activities, including proliferation, differentiation, and migration [21]. Impairment of tight junctions might have indicated severe gastrointestinal pathologies [22]. Prolonged exposure to HFD elevates oxidative stress, leading to a lack of tight junctions and significantly affecting the integrity of the gut epithelial barrier. However, there was no significant difference in the expression of ZO-1 and Occludin levels between the NHP40 and NHP80 groups. This is probably because NHP can exert a beneficial activity in promoting the expression of ZO-1 and Occludin, even at a relatively low concentration.

### 2.3. NHP Mitigated HFD-Induced Inflammation

**Figure 3:**
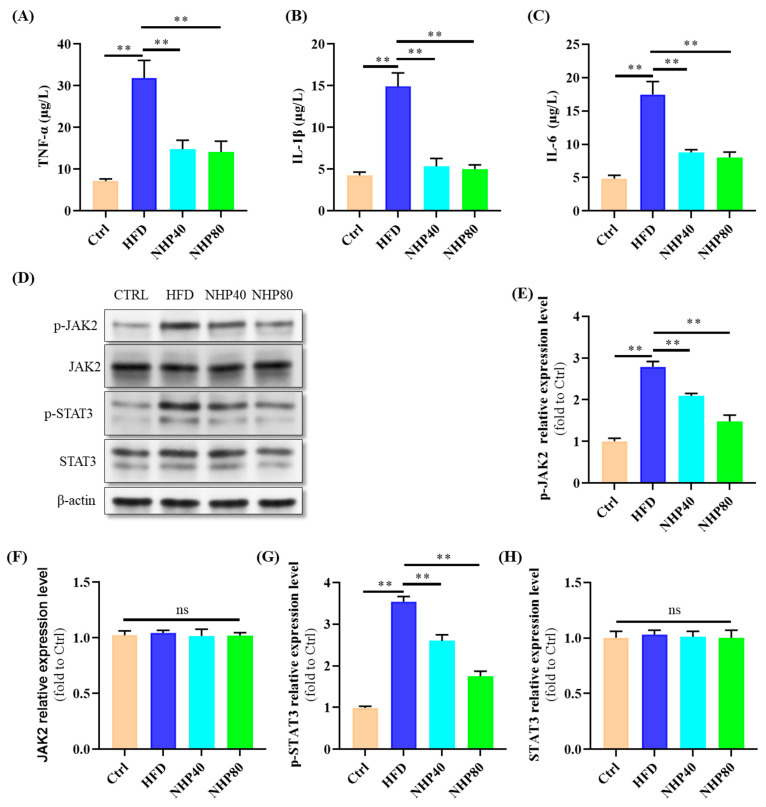
The level of tissue inflammation. The relative expression levels of (**A**) TNF-α, (**B**) IL-1β, and (**C**) IL-6. (**D**) Western blot results. (**E**–**H**) Quantification results. *n* = 3. ** *p* < 0.01, ns: no significant difference.

Figure 3A–C illustrate the potent anti-inflammatory efficacy of NHP, as evidenced by a significant reduction in the levels of TNF-α, IL-1β, and IL-6 (*p* < 0.01). Elevated circulating levels of TNF-α, IL-1β, and IL-6 are indicative of inflammation [23]. These findings suggest that NHP treatment attenuated HFD-induced inflammation. Figure 3D,E–H correspondingly reveal an amplification of JAK2/STAT3 signaling in HFD rats compared to Ctrl (*p* < 0.01), and dose-dependent suppression of p-JAK2 and p-STAT3 proteins by NHP was observed. Specifically, p-JAK2 protein expression decreased from 2.79-fold in the HFD group to 2.09-fold in NHP80 and 1.48-fold in NHP40, respectively. Similarly, p-STAT3 protein expression declined from 3.54-fold in the HFD group to 2.60-fold in NHP80 and 1.75-fold in NHP40, respectively. In conclusion, NHP effectively counteracted HFD-induced inflammation by activating the JAK2/STAT3 pathway. In a high-fat environment, excessive lipid accumulation occurs within cells. Such metabolic stress can cause certain intracellular stress signals to converge and activate the JAK2/STAT3 pathway. When activated, this pathway regulates the expression of many metabolic genes. For example, it may upregulate the expression of genes related to fatty acid synthesis, exacerbating intracellular lipid accumulation [24]. Consequently, the findings suggest that NHP reduces fat accumulation by reducing the activation of the JAK2/STAT3 pathway.

### 2.4. NHP Maintained the Integrity of Colonic Mucosa

**Figure 4:**
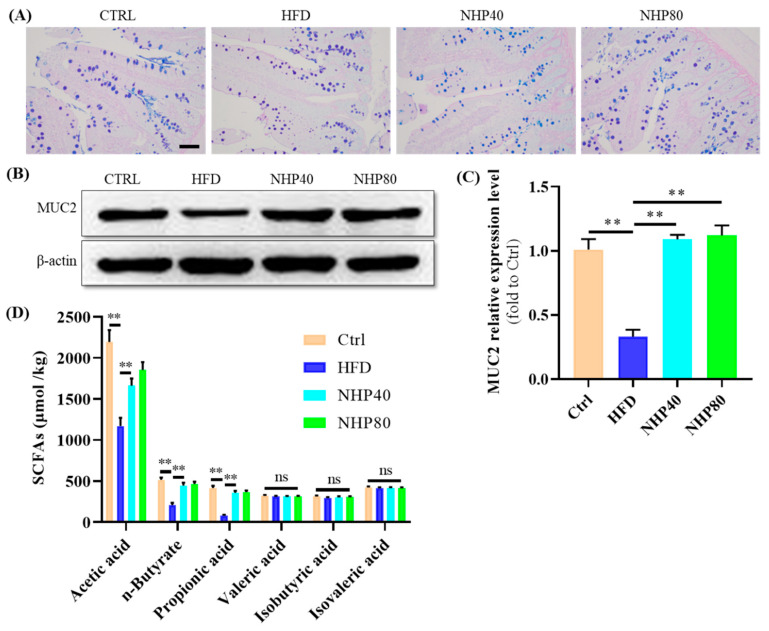
Analysis of AP-BAS staining, Western blot, and SCFA content. (**A**) AP-BAS staining results, in these images, blue areas represent acidic mucous substances (visualized by Alcian Blue staining), and the glycogen and neutral mucous substances are magenta (stained by Periodic Acid-Schiff). (**B**) Western blot results, (**C**) quantification results, and (**D**) content of SCFAs. The scale bar = 200 μm. *n* = 3. ** *p* < 0.01, ns: no significant difference.

In addition to its beneficial effects on lipid metabolism and inflammation, NHP also exhibited a role in maintaining the integrity of the colonic mucosa. Figure 4A shows a reduction in Alcian Blue-Periodic Acid-Schiff (AB-PAS)-stained areas during chronic HFD intake. NHP treatment counteracted HFD-induced depletion of goblet cells. Western blot analysis showed that the expression level of MUC2 protein in the ileal tissue of the HFD group was significantly reduced (Figure 4B,C). Quantitatively, it was found to be only 0.33 times that of the Ctrl group, with a statistically significant difference (*p* < 0.01). Interestingly, NHP significantly increased the relative expression of this protein (*p* < 0.01). AB-PAS staining is a widely used technique to detect intestinal and gastric mucous substances. In this method, glycogen and neutral mucous substances stain red, while acidic substances stain blue. The intestinal mucosal layer is vital in forming the mucosal barrier, with MUC2 being the primary mucin component. Thus, NHP mitigated HFD-induced colon mucosa damage by reducing inflammation. The mucosa layer of the intestine serves as an important barrier structure. When the intestinal mucosal barrier malfunctions, infections, toxic substances, and other dangerous agents can more easily penetrate the mucosal layer. Upon recognition of foreign infections by immune cells, an inflammatory reaction is initiated [5]. In this study, NHP treatment was found to reduce damage to the intestinal mucosal layer. By modulating certain molecular pathways, such as the JAK2/STAT3 pathway, NHP reduced inflammation, thereby mitigating the damage caused by HFD in colon mucosa.

### 2.5. NHP Elevated the Level of SCFAs

In Figure 4D, stool SCFA data indicated a significant decline in acetic acid, n-butyrate, and propionate levels among rats on HFD. After NHP supplementation, this downward trend in HFD-induced SCFA content was reversed, indicating that NHP treatment promoted SCFAs production. Acetic, propionic, and butyric acids, which are produced from carbohydrate digestion by gut bacteria, are rapidly utilized within humans [25]. Acetic acid has been found to enhance glucose tolerance and insulin release in overweight animals [26]. Butyric acid, which is essential for colon epithelial energy production, is beneficial in the fight against diabetes and insulin resistance [27]. Propionic acid, an essential gut metabolite, confers benefits to fat cells through anti-inflammatory effects, promoting adipocyte glucose utilization and lipogenesis [28]. Consequently, we suggest that NHP influenced SCFAs through gut microbiota regulation.

### 2.6. NHP Regulated Intestinal Flora Diversity

**Figure 5:**
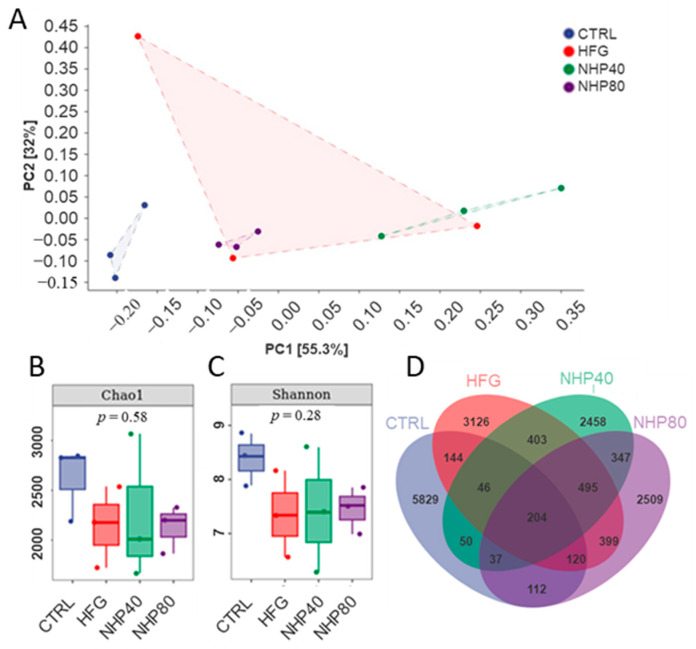
16S rDNA sequencing of bacterial communities. (**A**) PCA, (**B**) the Chao1 index, (**C**) the Shannon diversity index, and (**D**) ASV Venn diagram.

PCA analysis (Figure 5A) revealed the distinct positioning of Ctrl in the lower left corner, demonstrating a significant impact of HFD on gut flora. The NHP40 and NHP80 groups exhibited unique features from the HFD and Ctrl groups, suggesting partial restoration of HFD-induced alterations by NHP. Figure 5B,C illustrates a decrease in species richness and evenness in rats fed the HFD compared to Ctrl. However, NHP treatment did not significantly alter these parameters. As depicted in Figure 5D, NHP affected gut population composition. Shared amplicon sequence variants (ASVs) of 514, 337, and 473 were observed for Ctrl and HFD, Ctrl and NHP40, and Ctrl and NHP80, indicating NHP-induced shifts in gut microbiota composition. The Chao1 index represents species richness, and the Shannon index measures species evenness. After NHP treatment, there were no significant differences in Chao1 and Shannon gut microbiota indices. Based on the above results, we believe that NHP may play a regulatory role by regulating the spatial distribution, activity status, or metabolic activity status of the gut microbiota.

### 2.7. NHP Modulated the Overall Structure and Composition of Gut Microbiota

**Figure 6:**
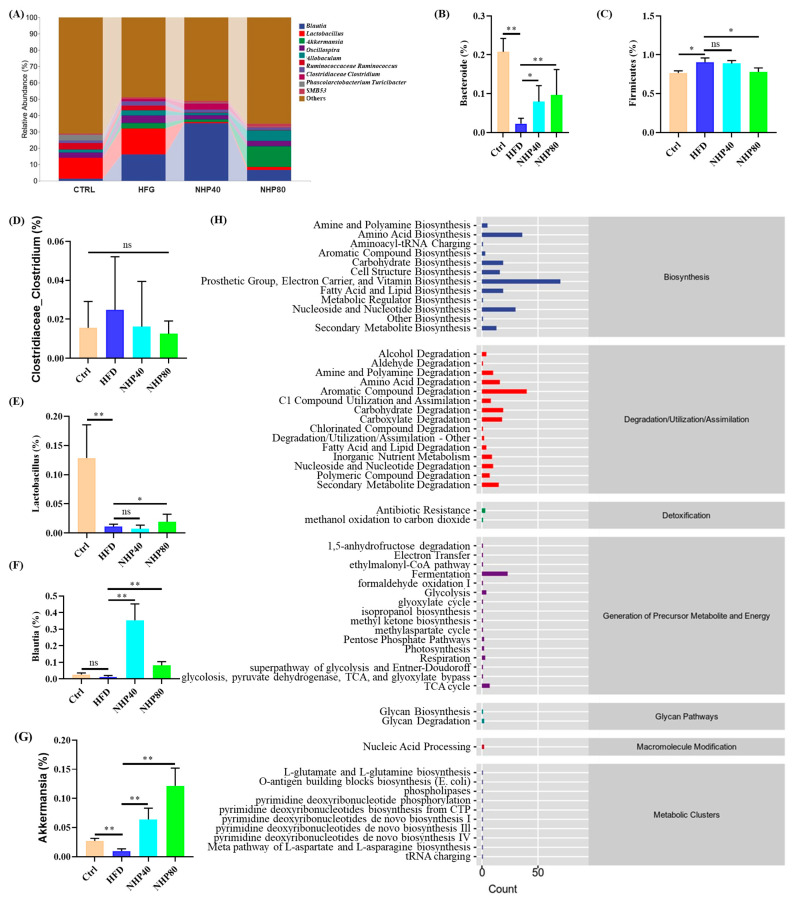
Bacterial composition and abundance in the gut microbiota. (**A**) Genus-level composition. Relative abundance of (**B**) *Bacteroide*, (**C**) Firmicutes, (**D**) *Clostridium*, (**E**) *Lactobacillus*, (**F**) *Blautia*, and (**G**) *Akkermansia*. (**H**) KEGG metabolic pathway comparison. *n* = 3. * *p* < 0.01, ** *p* < 0.01, ns: no significant difference.

At the genus level, the gut microbiota consisted mainly of genera including *Blautia*, *Lactobacillus*, *Akkermansia*, *Oscillospira*, *Allobaculum*, *Bacteroides*, *Clostridium*, *Phascolarctobacterium*, *Turicibacter*, and *SMB53* (Figure 6A). Compared to Ctrl, the proportion of Bacteroidetes decreased significantly in HFD (*p* < 0.01) (Figure 6B), while Firmicutes increased substantially (*p* < 0.05) (Figure 6C). This indicates that HFD treatment notably enhanced the Firmicutes/Bacteroidetes (F/B) ratio. Administration of NHP resulted in a significant increase in Bacteroidetes and a reduction in Firmicutes proportions in a dose-dependent manner (*p* < 0.05). Previous studies have shown that an elevated F/B ratio in the HFD group is associated with nutrient accumulation, adipogenesis, and fat storage [29]. Thus, the reduced F/B ratio may be related to NHP’s protective role against glycolipid metabolism disorders. As shown in Figure 6D, there was no significant difference in the abundance of *Clostridium* levels among each group (*p* > 0.05). *Lactobacillus*, which produces lactic acid and plays a crucial role in maintaining the intestinal mucosal barrier, regulating the immune system, and promoting digestion and absorption [30], exhibited a significant increase in abundance in the NHP80 group (*p* < 0.05) (Figure 6E). Research by Hosimi et al. [31] has shown that higher levels of *Blautia* suggest a smaller visceral fat area. In vitro and in vivo experiments have also confirmed *Blautia*’s ability to reduce BW gain, inflammation, and insulin resistance [32]. Consequently, this bacterium has emerged as a key obesity-related gut bacterium [31]. In this study, NHP treatment significantly increased *Blautia* abundance (Figure 6F). The increase in beneficial bacteria such as *Blautia* may contribute to improved lipid metabolism through mechanisms such as enhanced bile acid metabolism and reduced inflammation-induced insulin resistance. A low level of *Akkermansia* in the intestine may lead to thinning of the mucosa, leading to a weakening of the intestinal barrier function and making it easier for toxins to invade the host [33]. Fortunately, after NHP treatment, the level of this bacterium increased significantly (Ctrl: 0.027%, HFD: 0.009%, NHP40: 0.064%, NHP80: 0.122%, *p* < 0.01) (Figure 6G). To clarify the potential mechanism by which the gut microbiota affects the organism, this study used PICRUSt2 to perform functional sequencing on the results of abundance of all samples in secondary pathways. Results (Figure 6H) reveal that the differential gut microbiota focused primarily on biosynthesis, particularly amine, polyamine, fatty acid, nucleotide, and cofactor biosynthesis. In addition, previous studies [14,16,34] have shown that polyamines exert effects on renewing and repairing intestinal epithelial cells and maintaining the integrity of the intestinal barrier. Meanwhile, fatty acids play a crucial role in maintaining the function of the intestinal barrier. These findings suggest that NHP effectively alleviates elevated HFD-induced inflammation and potentially preserves the structural integrity of intestinal epithelial tissue by modifying the gut microbiota.

### 2.8. In Vitro Fermentation Results

#### 2.8.1. Fermentation pH Fluctuation

**Figure 7:**
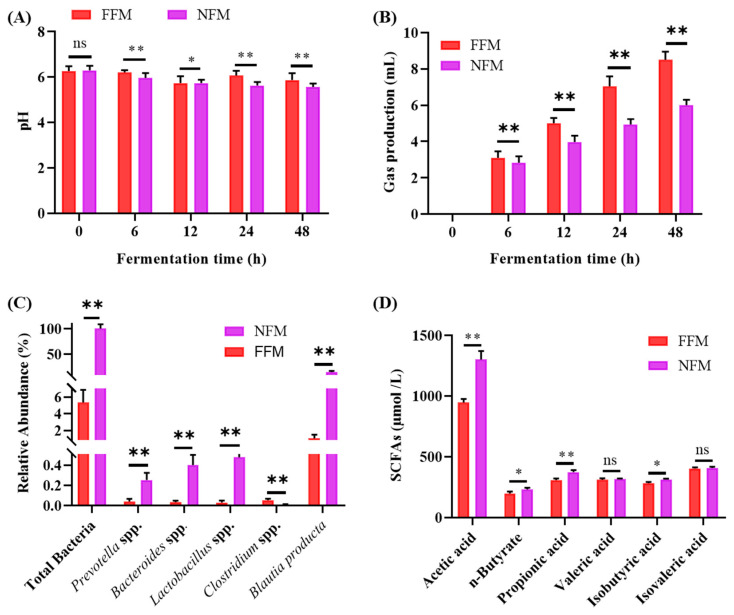
In vitro fermentation culture results. (**A**) pH change, (**B**) gas production, (**C**) SCFAs concentrations, and (**D**) intestinal microbiota composition in fermentation cultures. *n* = 3. * *p* < 0.01, ** *p* < 0.01, ns: no significant difference.

As shown in Figure 7A, the pH values of fecal samples from both donor rats of HFD (FFM) or NHP80 (NFM) decreased gradually over the 0–48 h fermentation period. Specifically, the pH value of FFM dropped from 6.27 at 0 h to 5.87 at 48 h, while the pH value of NFM decreased from 6.30 to 5.57. Research [35] has shown that an appropriate decrease in the pH of gut lumen can provide beneficial conditions for the growth of beneficial colonic microbiota and inhibit the growth of harmful microorganisms.

#### 2.8.2. Fermentation Gas Production

As illustrated in Figure 7B, gas production increased steadily as fermentation progressed. The FFM group produced 8.5 mL of gas over 48 h. NFM gas production was 6.0 mL. Carbohydrates and proteins will be hydrolyzed by colonic flora, giving rise to gases such as carbon dioxide, hydrogen, and methane. Although certain gases facilitate intestinal peristalsis, excessive gas can lead to adverse consequences such as abdominal distension and appetite suppression [36].

#### 2.8.3. Microbiota Composition and SCFAs Production

Quantification of bacterial populations in the fermentation broth revealed an increase in total bacteria as well as abundance of *Prevotella*, *Bacteroides*, *Lactobacillus*, and *Blautia* in NFM group. In contrast, *Clostridium* populations decreased significantly in the FFM group (*p* < 0.01) (Figure 7C). These bacterial genera are well-known producers of SCFAs [37]. *Prevotella*, for example, contributes significantly to carbohydrate degradation and produces acetic and propionic acids. *Bacteroides* ferments dietary fiber to produce acetic, propionic, and butyric acids. *Lactobacillus* produces lactic acid, which is further converted into SCFAs by other gut bacteria [37,38,39]. After 48 h of fermentation, significant differences in specific SCFAs were observed between the two groups (*p* < 0.05, *p* < 0.05) (Figure 7D). Specifically, the levels of acetic acid, n-butyrate, propionic acid, and isobutyric acid in the NFM group were 1300, 231, 373, and 314 μmol/L, respectively, which were significantly higher than those in the FFM group (945, 197, 308, and 282 μmol/L). In conclusion, NHP treatment increased the abundance of bacteria capable of producing SCFAs in the feces of experimental rats. However, this study has certain limitations. For example, the long-term effects of NHP treatment need to be further investigated.

## 3. Materials and Methods

### 3.1. Experimental Materials

NHP was acquired from Kang Biotech (Lianyuan, China). The DNeasy PowerSoil Kit (Cat #12888-100) was purchased from Qiagen (Hilden, Germany). Q5^®^ High-Fidelity DNA Polymerase (Cat #M0491L) was purchased from NEB (Ipswich, MA, USA). The double-stranded DNA quantitative assay kit (Cat #P7589) was procured from Invitrogen (Carlsbad, CA, USA). Agencourt AMPure XP (Cat #A63881) was purchased from Beckman Coulter (Pasadena, CA, USA). NovaSeq 6000 SP Reagent Kit (Cat #20028402) was purchased from Illumina (San Diego, CA, USA). AXYGEN DNA Gel Recovery Kit (Cat #AP-GX-50G) was purchased from Axygen biosciences (Union City, CA, USA). Acetic acid, propionic acid, n-butyrate, valeric acid, isobutyric acid, and isovaleric acid were all procured from Sigma-Aldrich (St. Louis, MO, USA).

### 3.2. Experimental Animals and Treatments

In this study, the 32 experimental animals were Sprague-Dawley male rats aged 5–6 w and weighing 180–220 g, specific pathogen-free (SPF)-qualified, from SJA, in Changsha, China. They were housed in a SPF environmental condition, and the incubation ambient conditions were maintained at a temperature range of 20 to 26 °C, with humidity ranging from 40% to 70% and a fixed 12 h light/12 h dark cycle. Throughout the experimental period, all rodents received unrestricted access to water and food. Laboratory animal experiments were carried out at the Institute of Laboratory Animal Research (Hunan University of Chinese Medicine). Laboratory rodent diets, including high-fat and basic types, were purchased from BKF Co., Ltd. (Beijing, China). Following one-week adaptation, rats were divided into four equal-sized groups with eight animals in each group, including a blank control group (Ctrl, fed basic laboratory rodent diet), HFD model group (HFD, high-fat laboratory rodent diet), 40 mg/kg BW NHP treatment group (NHP40, fed HFD and intragastric administration of 40 mg NHP/kg BW per day), and 80 mg/kg BW NHP treatment group (NHP80, fed HFD and intragastric administration of 80 mg NHP/kg BW per day). The doses of 40 mg/kg BW and 80 mg/kg BW were selected based on a previous study [40] and preliminary experiments in our laboratory, which showed significant effects on relevant indicators. For administration, NHP was reintegrated into a 0.5% sodium carboxymethylcellulose (CMC-Na) environment. An equivalent dose of CMC-Na gel was provided daily to both Ctrl and HFD NHP-free groups over a 12-week period. Biweekly BW and weekly diet intake assessments were performed. After treatment, the animals were humanely euthanized with chloral hydrate, along with intestinal contents, blood serums, and liver collections.

### 3.3. Colorectal Histopathological Examination

Colorectal tissue was subjected to H&E or AB-PAS staining to display histopathological characteristics. Specifically, colon segments preserved in formalin or Carnoy’s solution were subjected to H&E or AB-PAS staining, respectively. After a 24 h fixation period, colonic tissue was dehydrated, paraffin-embedded, and segmented into thin slices. Afterwards, they were treated with H&E or AB-PAS. Pathological assessments were carried out using an BH2 microscope (Olympus, Hino, Japan) at a magnification of 200×.

### 3.4. Immunohistochemical Detection

After deparaffinization, tissue sections were accurately adhered to gelatinized slide chambers. These slides were then subjected to initial immune staining with anti-ZO-1 (Cat# 21773-1-AP), anti-Occludin (Cat# 13409-1-AP), or anti-MUC2 (Cat# 27675-1-AP) from Proteintech (Wuhan, China). A rinse with phosphate-buffered saline (PBS) was performed prior to application of HRP-tagged secondary antibody (Abcam’s Cat# ab205718, Cambridge, United Kingdom). Subsequently, DAB staining (Sigma-Aldrich, St. Louis, MO, USA) and hematoxylin counterstaining were carried out to facilitate the assessment of immunoreactivity using the H-score methodology. In this methodology, scores of 1, 2, and 3 were assigned for minimal, moderate, and strong staining, respectively. The H-score was then calculated as 1 × %minimal + 2 × %moderate + 3 × %strong.

### 3.5. Cytokine Analysis

Twelve weeks post-intervention, nocturnal fasting and subsequent anesthesia were applied to rodent subjects. Three or four rats were randomly selected for blood collection from the left ventricle. The biochemical values of TC, TG, HDL-C, LDL-C, and liver enzymes AST and ALT were evaluated using Jiancheng Bio (Nanjing, China) kits. The serum cytokine levels of TNF-α, IL-1β, IL-6, IL-10, and IL-17 were quantified by ELISA from Bio-Swamp (Wuhan, Hubei, China) in accordance with the specified protocol. Absorption readings at 450 nm were obtained using a MK3 microplate reader from LabSystems (Vantaa, Tiolitie, Finland).

### 3.6. Intestinal Microbiota Sequencing Procedure

Intestinal genomic DNA was isolated using Qiagen’s DNeasy PowerSoil Kit and evaluated with Nanodrop ND-1000 (Thermo Fisher, Waltham, MA, USA). Conventional agarose gel electrophoresis was employed for further verification. The V3-V4 region of the 16S RNA gene was amplified by 338F and 806R primers. Amplified products were purified with Agencourt AMPure beads and quantified by PicoGreen (Thermo Fisher, Waltham, MA, USA). The MiSeq platform generated up to 2 × 300 bp sequences per sample using Illumina’s MiSeq Reagent Kit v3 (Illumina, San Diego, CA, USA). After quality control, sequences with 97% similarity formed operational taxonomic units (OTUs). Analyses focused on α diversity (Chao1 and Shannon indices) and β diversity (PCAs). Visualization was assisted by PCoA plots in MEGA 6.13.1 and GraPhlAn 1.1.3. A Venn diagram in the R package (v4.2.3) illustrated shared and unique OTUs across samples/clusters. The heatmap clustering and KEGG analysis were carried out on the Pannoramix cloud platform (www.genescloud.cn, accessed on 24 December 2023).

### 3.7. Fecal Microbiota In Vitro Fermentation

Three fecal samples from donor rats of HFD (FFM) or NHP80 (NFM) were collected under aseptic conditions. Diluted to 20% (*w*/*w*) with Dulbecco phosphate buffer, samples underwent 10,000 rpm homogenization for 1 min and filtration through aseptic gauze. According to the protocol by Bianchi et al. [41], a 1.0 L medium was prepared, which contained 5.0 g each of soluble starch, peptone, and tryptone; 4.5 g each of yeast extract, NaCl, and KCl; 4.0 g of mucin; 3.0 g of casein; 2.0 g of pectin; 2.0 g of arabinogalactan; 1.5 g of NaHCO_3_; 1.0 g of guar; 0.8 g of L-cysteine HCl-H_2_O; 0.69 g of MgSO_4_-H_2_O; 0.5 g of KH_2_PO_4_; 0.5 g of K_2_HPO_4_; 0.4 g of bile salt; 0.08 g of CaCl_2_; 0.005 g of FeSO_4_-7H_2_O; 1.0 mL of Tween 80; and 4.0 mL of 0.025% resazurin solution. This medium was autoclaved at 121 °C for 15 min before being distributed into sterile glass vessels. A ratio of 1.6 mL medium to 0.4 mL bacterial suspension was used. The mixture was transferred to an anaerobic LAI-3 incubator (Longyue Instrument Corp., Shanghai, China) for shaking culture at 37 °C, humidity 75%, and a gas composition of CO_2_ (80%), N_2_ (10%), and H_2_ (10%). The shaker operated horizontally at a speed of 250 rpm. All experiments were repeated thrice.

### 3.8. Measurement of pH and Gas Production

Fermentation was terminated by immersing in ice for 5 min. Centrifugation was then performed at 8000 rpm for 15 min to efficiently isolate the supernatant. The pH of these product solutions was quantified using a pHS-3C pH meter (Leici, Shanghai, China) after centrifugation. For gas production measurement during fermentation, a 10 mL disposable syringe was used. The needle tip of the syringe was inserted into the tube through the rubber stopper at the upper end of the anaerobic tube. The height of the raised syringe piston was used to determine gas production during the fermentation process.

### 3.9. Determination of SCFAs Content

SCFAs quantification involved centrifugation at 8000 rpm for 15 min followed by filtration with a 0.22 μm filter. Analysis was executed using the ISQ-LT GC-MS system (Thermo Fisher, Waltham, MA, USA) with a TG-WAX column (30 m × 0.25 mm × 0.25 μm). The initial column temperature was 140 °C for 6 min and then increased to 160 °C at 5 °C/min for another 6 min. Auxiliary injection and MS source temperatures were set at 180 °C and 200 °C, respectively. Nitrogen flow rate was 0.8 mL/min, with an injection volume of 1 μL (split ratio of 20:1). Individual SCFA levels were derived from corresponding standard curves.

### 3.10. RT-qPCR Detection of Specific Bacterial Groups

Total RNA was isolated using Ambion TRIzol reagent (Cat#: 15596026, Foster City, CA, USA). Subsequently, cDNA conversion was performed with a Clontech Advantage RT-PCR Kit (Cat#: 639505, Palo Alto, CA, USA). Real-time qPCR reactions were performed with Kapa Biosystems SYBR Green Master Mix (Cat#: KM4101, Wilmington, MA, USA) on a Bio-Rad real-time PCR system (Hercules, CA, USA) for 39 cycles. Primers for RT-PCR (sequences provided in Table 1) were custom-synthesized by GenScript Biotech Corp (Nanjing, Jiangsu, China). Bacterial abundance was normalized against total bacterial counts.

### 3.11. Western Blot Analysis

Total tissue protein from the colon was extracted by RIPA lysate and assayed by BCA protocol. Proteins were subjected to electrophoresis and then transferred to membranes. Subsequently, the membranes were blocked with 5% skim milk powder for one hour. Overnight incubation at 4 °C with primary antibodies was carried out, followed by three washes with TBST. A secondary antibody dilution of 1:5000 was added for one hour, followed by additional TBST washes. After adding a luminescent liquid, the membranes were imaged by a gel imaging system. Protein expression levels were assessed using Image J 1.8.0 software.

### 3.12. Statistical Methods

Statistical evaluations and figure generation were performed using Prism 8.0 (GraphPad, La Jolla, CA, USA). For specific tasks, one-way analysis of variance (ANOVA) with Tukey post hoc analysis or Student *t*-test was employed. Post hoc tests were utilized for significant overall effects (two-way or one-way). All statistical computations were carried out using SPSS Statistics 30. The significance threshold was defined as *p* < 0.05.

## 4. Conclusions

NHP has shown substantial efficacy in fighting HFD-induced inflammation by modulating gut microbiome composition. Specifically, it influences Firmicutes ratios and promotes the growth of beneficial species such as *Bacteroides*, *Blautia*, *Lactobacillus*, and *Akkermansia*. These adjustments mainly resulted in increased production of SCFAs. In conclusion, 40–80 mg/kg BW NHP has demonstrated significant efficacy in alleviating HFD-induced inflammation by modulating gut microbiota composition and promoting SCFAs synthesis. In the present study, although NHP40 and NHP80 exhibited similar tendencies in improving lipid metabolism disorders, alleviating pathological ileum injury, mitigating HFD-induced inflammation, maintaining colonic mucosa integrity, enhancing SCFAs levels, and regulating intestinal flora diversity, more significant alterations were observed in serum AST and HDL-C levels in experimental animals, as well as the abundance of Firmicutes, *Lactobacillus*, and *Akkermansia* in the intestinal flora after NHP80 treatment. Although ZO-1 had a higher relative abundance in NHP40 than in NHP80, this could be attributed to the study’s small sample size. Therefore, we believe that 80 mg/kg BW NHP has the potential to prevent and treat HFD-related diseases. Future studies should focus on translating these results into clinical applications and exploring the optimal dosage and treatment duration.

## Figures and Tables

**Table 1:** Primer sequence information.

	Forward (5′-3′)	Reverse (5′-3′)
Total bacteria	TCCTACGGGAGGCAGCAGTGE	TTACCGCGGCTGCTGGCACG
*Prevotella*	CGGTGAATACGTTCYCGG	GGWTACCTTGTTACGACTT
*Bacteroides*	GGTGTCGGCTTAAGTGCCAT	GCATTYCACCGCTACACATG
*Lactobacillus*	GCAGCAGTAGGGAATCTTCCA	GCATTYCACCGCTACACATG
*Clostridium*	AGAGTTTGATCCTGGCTCAG	ACGGCTACCTTGTTACGACTT
*Blautia producta*	AGCTGACGACCTGATCGAGT	TCTCGAGCTGGTACGCTTCA

## Data Availability

The data generated in the present study may be requested from the corresponding author.
